# Pancreatogenic diabetes pacient selection for pancreatic islet transplantation

**Published:** 2010-02-25

**Authors:** C Grigore, V Sarbu, S Simion, I Simion, T Poteca, S Nedelea

**Affiliations:** * The 1^st^ Surgery Department of Colentina Clinical Hospital, BucharestRomania; ** The Surgery Department of the Clinical Emergency County Hospital, ConstantaRomania; *** ‘Carol Davila’ University of Medicine and Pharmacy, BucharestRomania

**Keywords:** pancreatectomy, HbA1C, indications, contraindications

## Abstract

Background–recently, insulin–dependent diabetes mellitus, can be treated by pancreatic islet allotransplantation.

Methods–This retrospective study involves 137 patients from the Surgery Department of Colentina Clinical Hospital, Bucharest, in the July 2000–July 2008 period, who underwent pancreatic resections, the number of patients who developed pancreatogenic diabetes and their selection for the pancreatic islet transplantation.

Results–After pancreatectomy, 70 patients are diagnosed with diabetes, and 42 with prediabetic stages (IFG and IGT). 61 of these had average glycemic excursions (MAGE) over the normal, and 31 of the 70 patients diagnosed with diabetes, presented hypoglycemic episodes during treatment.

Conclusion–The present criteria of patient selection for pancreatic islets transplantation are limited and can be applied to a small number of patients.

## Introduction

Insulin–dependent diabetes can be treated by complex dietary measures and insulin administration, and more recently by pancreatic/pancreatic islets allotransplantation. [1]

The whole pancreas transplantation encompasses two categories of major risks and complications:

1. the immunosuppressive treatment;
2. the risk of major surgery in patients with a chronic condition.

Pancreatic islets transplantation seems more reasonable than the whole pancreas transplantation. It demands a shorter surgical procedure, involving less invasive methods of anesthesia for the body, but requires a very complicated technology of preparing the pancreatic islets. [2]

Therefore, the risks and complications of immunosuppressant treatment currently remain research heads in developing microcapsules for pancreatic islet transplantation that do not require an immunosuppressant treatment. The permeability properties of these microcapsules allow the penetration of nutrients and glucose and elimination of insulin secreted by pancreatic islet.[3]

Moreover, current studies suggest that the actual number of beneficiaries of a possible pancreatic islet transplant is very low. For this reason, research is headed to stem cells use for diabetes therapy because of their multipotency and recent applications in the treatment of serious diseases (Alzheimer's disease, cardiac pathology, etc.).[4]

## Objectives

This paper is elaborated on the pancreatogenic diabetes patient selection criteria for the pancreatic islet transplantation.

## Materials and methods

This study involves 137 patients from the Surgery Department of ‘Colentina’ Clinical Hospital, Bucharest, from July 2000 to July 2008.

Inclusion criteria: age above 18 years old, known pancreatic pathology (acute/chronic pancreatitis, pancreatic cancer)

Exclusion criteria: patients known to suffer from acute/chronic pancreatitis and pancreatic cancer, who did not undergo pancreatic resection.

The main indication for surgery was the lack of response to pain medication, eating disorders, weight loss, repeated periods of hospitalization, decreased work capacity and imagistic evidence of morphologicalchanges of the pancreas.

Only subtotal pancreatectomies were conducted in this study.

The variables in this study are represented by the indications/contraindications in the pancreatic islet transplantation stated by the Edmonton protocol.

Indications for pancreatic islet transplantation (According to the Edmonton protocol) [5]

- Patients with type Ⅰ diabetes aged between 18 and 65 years old who have been diagnosed with diabetes for 5 years;
- Severe hypoglycemic answers;
- Unstable diabetes:
  1. Metabolic instability sufficient to cause the worsening of the patient's lifestyle and endangering his life, even if the patient uses a strict insulin administration schedule and blood glucose monitoring is done 4 times a day
  2. Metabolic instability manifested by chaotic blood glucose profile
  3. Number of hypoglycemic episodes or ketoacidosis (two episodes that required assistance with hospitalization for hypo or hyperglycemia in the last 12 months)
  4. Increase in mean amplitude glycemic excursions (MAGE) (> 6.6 mmol / l (118.8 mg / dl) while normally it is <3.5 mmol/l (63mg/dl))
  5. Altered way of life, determined by the number of hospital admissions per year (two or more), the absence from work or school (4 weeks or longer), or the inability to cope with everything, alone at home or in another environment
- Reversible secondary diabetic complications:
  1. progressive microalbuminuria receive treatment (proteinuria <3g/day) even on ACE inhibitors
  2. difficulties given by documented autonomic/peripheral neuropathy
  3. documented proliferative retinopathy
- negative C peptide (<0.2 ng/ml after iv administration of 5 grams of arginine)
- Doses of insulin per day <0.7 U/per kg of bodyweight/day)

Contraindication for pancreatic islet transplantation [5]

- severe coexisting cardiac disease:
  1. myocardial infarction–in the last 6 months
  2. angiographically record of irreversible CAD (coronary artery disease)
  3. cardiac ejection fraction <40%
- Alcohol or other substance abuse, including smoking (to be stopped 6 months before transplant)
- Major psychiatric disorders
- Active infections: hepatitis C, hepatitis B, HIV positive,  Mantoux test (skin reaction to PPD)
- History of malignancy (unless they have a free interval of at least 5 years)
- Portal hypertension
- Body weight index over 26, or weight > 70 kg in women and > 75 kg in men
- C peptide > 0.2 ng / ml
- Age below 18 or more than 65 years old
- Creatinine clearence <60 ml/min/1, 73 meters
- History of non–compliance to medication, including immunosuppressive
- HbA1c > 10%
- Untreated proliferative retinopathy
- Positive pregnancy test
- Uncontrolled hyperlipidemia
- Diseases that require chronic administration of steroids
- Symptomatic biliary lithiasis
- Coagulopathy or conditions requiring long–term administration of anticoagulant therapy

Patients with insulin–dependent diabetes who address to transplant teams are registered on waiting lists in the following situations:

1. If they have both diabetes and concomitant chronic renal failure and are candidates for a kidney transplant, and can thus solve both conditions at the same time, or in turns (double transplant / post renal transplant).[6]
2. On grounds of benign pancreatic disease severity, they undergo a total or subtotal pancreatectomy. Most frequently, this happens in severe chronic pancreatitis, but also in some pancreatic trauma.
3. In patients with type Ⅰ diabetes (hypoglycemia, coma, etc.) difficult to control

## Results

Patient' distribution by admission time and pathology.

Of the patients who required pancreatectomy during July 2000–July 2008, 23.35% of them were diagnosed with acute pancreatitis, 47.45% with chronic pancreatitis and 29.2% with pancreatic cancer.

Although patients who have undergone a pancreatectomy for pancreatic cancer, would fit in terms of postoperative diabetes in the selection criteria for pancreatic islet transplantation of pancreatic islands, the very disease they had surgery for, excludes them. [Table 1]

**Table 1:** Distribution by hospital admission year and diagnosis

Admission year	Diagnosed with acute pancreatitis	Diagnosed with chronic pancreatitis	Diagnosed with pancreatic cancer	Diagnosed with pancreatic cancer
2001	5	6	4	15
2002	4	9	6	19
2003	3	9	4	16
2004	4	8	7	19
2005	4	7	6	17
2006	5	10	3	18
2007	3	7	6	16
2008	4	9	4	17
Total	32	65	40	137

### Age at hospital admission

**Fig 1:**
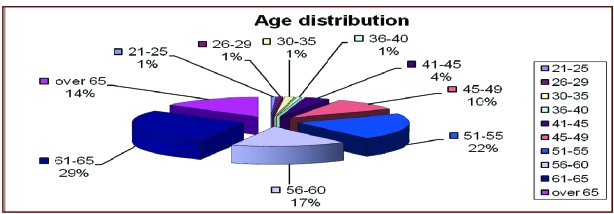
Age distribution

The chart below shows that most of the patients in the study group are aged between 45 and 65 years old. Roughly, there are 2 times more males than females. [Fig 1]

### Blood glucose levels

**Fig 2:**
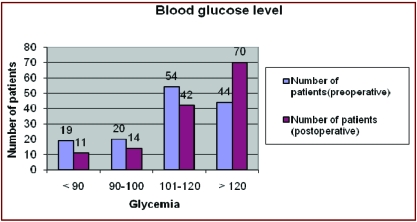
Blood glucose level

Fasting blood glucose levels were monitored in all patients during hospitalization. As it is clear from the evidence presented, the number of patients with blood glucose values over 120 rose significantly from 44 patients preoperatively, to 70 patients postoperatively, constituting 51.09% of all patients. The 42 patients with postoperative blood glucose levels between 101 and 120 mg / dl (IFG–impaired fasting glucose) had glucose tolerance test after surgery. Two hours after glucose ingestion 34 of them had blood glucose values greater than 140mg/dl. They were diagnosed with IGT (impaired glucose tolerance), prediabetic stage that should be kept under observation. [Fig 2] 

Patients with diabetes and prediabetic stages (IFG,IGT) had their mean glycemic excursions (MAGE) tracked. 61 of the 112 patients (50% of total) had mean values that exceeded 3mg/dl (value considered standard).

### Hospital stay

**Fig 3:**
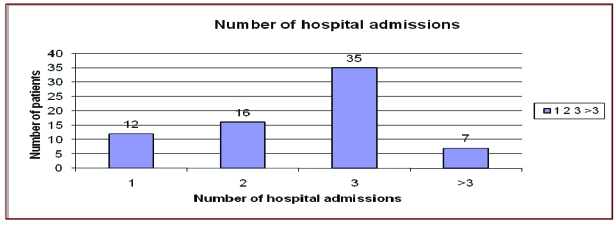
Number of hospital admissions

The average length of hospitalization of patients with no complications was of 10 days (range: 8–14 days) and for those that had complications, the average length of stay was of 41 days (range: 32–50 days). None of the patients who underwent further surgery required distal pancreatic stump resection.[Fig 3]

Patients altered their lifestyle postoperatively due to long periods of hospital stay, along with sick leave, eating restraints and postoperative drugs. The number of yearly admissions to hospital (two or more), or absence from work /school (for 4 weeks or longer), or the inability to cope with everything alone at home or in another environment contributed to lifestyle alteration too. 58 patients (74.28% of the total 70 diagnosed with diabetes) had transplant indication due to their postoperative altered lifestyle.

### Unstable diabetes

18 out of the 70 patients postoperatively diagnosed with diabetes, had characteristics of unstable diabetes like: life threatening metabolic instability, even if patient was under insulin treatment and blood glucose monitoring was done 4 times per day. Metabolic instability manifested by chaotic blood glucose levels and episodes of hypoglycemia or ketoacidosis (two episodes that required assistance with hospitalization for hypo/ hyperglycemia in the last 12 months). A total of 32 patients experienced severe hypoglycemic responses, constituting 45.71% of the total of 70 patients with diabetes. 

Neurological and ophthalmologic examinations for early detection of potentially reversible secondary complications of diabetes were performed postoperatively during hospitalization in ‘Colentina’ Clinical hospital.

**Autonomous or peripheral neuropathy** (indication for pancreatic islet transplantation) was diagnosed in 16 patients (22.85% of the postoperative diabetic patients), with equal sex distribution. It is the most common form of diabetic neuropathy.

**Autonomic neuropathy** (autonomic) results from damage to the autonomic nervous system. These nerves are involved in involuntary body functions such as heartbeat, blood pressure, perspiration, digestion, kidney function and some aspects of sexual function. This is a common form of diabetic neuropathy.

**Focal neuropathy** affects a single nerve, most commonly in the wrist, thigh or leg. It may also affect nerves in the back and anterior thorax and those that control eye muscles. Carpal tunnel syndrome often occurs in people who have diabetes but not focal neuropathy. Focal neuropathy usually appears suddenly and it is the least frequent form of diabetic neuropathy. [7] 

There is no recommended protocol for screening autonomous or focal neuropathy, but during regular medical examination multiple signs and symptoms should be alarming: pain of any intensity and localization, weakness or motor disorder, changes in digestion, kidney function or sexual behavior, sweating or dizziness.[7]

There is no cure for diabetic neuropathy. Once installed, the treatment focuses on secondary prevention (removal of precipitating factors) consisting of keeping blood glucose levels in a certain target. A tight control of blood glucose is to maintain an average glycosylated hemoglobin [HbA1c] under 7%, for a period of 2 to 3 months. 

**Microalbuminuria** is an important independent risk marker for proliferative diabetic retinopathy, as well as for hypertension. Microalbuminuria over 100mg/24h is associated with progressive decrease of glomerular filtration rate (by 3–4 ml/min/year), and is strongly predictive for the occurrence of proteinuria and chronic renal failure [8]. 10 out of 14 patients discovered with microalbuminuria during the study, developed proteinuria (300–3000g/day), necessary for the patient to have treatment indication, but 8 of the 14 patients had creatinin a clearance level of <60 ml/min/1,73m, this being among the contraindications. Pancreatic islet transplantation is specifically indicated in patients with type I diabetes who have a functional renal graft and are already immunosuppressed.

**Diabetic retinopathy** was diagnosed by the ophthalmologist in seven patients (10%), 5 of them having no previous treatment for the condition. Proliferative retinopathy is an indication of pancreatic islet transplantation, but untreated becomes a contraindication. [8]. Islet secretory function was also assessed by the determination of C peptide and glycosylated hemoglobin HbA1c. 

**C peptide** is a 31 amino acids chain that links the A and B chains of insulin in the proinsulin molecule. 42 patients had postoperative levels of C peptide above 0.2 ng/ml. C peptide above 0.2ng/ml is among the contraindications of the procedure.

**Fig 4:**
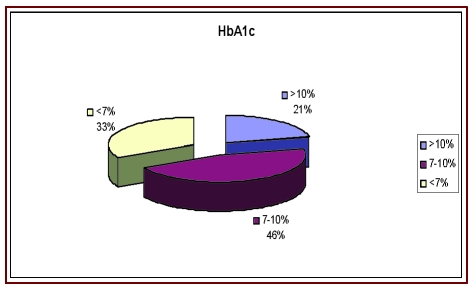
HbA 1C

**Glycosylated hemoglobin **– the normal maximum is 2.2–5%, but the goal in diabetes is to keep it under 7%. HbA1C determination has become mandatory in assessing therapeutic efficiency and the degree of metabolic balance. HbA1C has given important information in blood glucose levels in the past three months. Unlike a determination of glucose, which shows blood glucose level at a certain time and thus may mislead us, HbA1C shows a history of blood glucose levels, therefore it is known as ‘glucose memory’. [Fig 4] 

From the 70 patients with postoperative diabetes, 15 (21.42%) had postoperative HbA1c values > 10%, this value representing a contraindication.

Active ethanol abuse, as well as other substance abuse and smoking (which should be stopped 6 months before transplantation), were correlated with a history of non–compliance to medication. Patient compliance is very important due to the postoperative permanent immunosuppressive treatment. These factors are among the contraindications of the process and were encountered in 39 patients (55.71% of the total of 70 patients).

Diseases requiring long-term administration of anticoagulant therapy are also a contraindication. In this study, we encountered deep recurrent thromboembolism in seven patients (10%), atrial fibrillation in 8 patients (11.42%) and one case of ocular central vein thrombosis, (1.42%). Three patients (4.28%) required chronic administration of steroids, one with asthma and two with rheumatoid arthritis.

There were no cases of HIV infection or positive Mantoux test (skin reaction to PPD). Hepatitis B (31 patients–44.28%) and hepatitis C (9 patients–12, 85%) were the active infections encountered in these patients.Major psychiatric disorders, part of the contraindications of the procedure (organic personality disorder, schizophrenia, paranoid disorder) were detected in five of the 70 patients (7.14%). 

20% of the patients (14 patients) had treatment–resistant hyperlipidemia and 43 patients (61.42%) a body mass index of (BMI)> 26.Severe cardiac conditions are a contraindication: myocardial infarction in the last 6 months –3 patients (4.28%), angiographically evidenced coronary artery disease–22 patients (31.42%), cardiac ejection fraction <40% –9 patients (12.85%). Portal hypertension was diagnosed in 27 patients (38.57% of the studied group). No other malignancies were present and 28 patients (40%) had a concomitant colecistectomy.

## Conclusion

Given these results, we conclude that the selection criteria of patients are limitative. Pancreatic islet transplantation indications and contraindications should be reviewed or medical research interest should move toward stem cells use, in order to establish a better treatment for pancreatogenic diabetes. 
